# Glad Tidings

**Published:** 1876

**Authors:** 


*Glad Tidings.*—To those of our readers who have desired to know what had become of our distinguished associate editor, Dr. Alban L. Payne, of Virginia, we rejoice to say is rapidly recovering from a feeble state of health; that alone has prevented him from doing his part in sustaining THE RECORD. He promises to forward, as soon as possible, the long expected article that he was requested to prepare on typhoid pneumonia. We need not say that it will be eagerly looked for, and when it appears that it will be read by all with great pleasure and profit

WE will acknowledge receipt of dues for 1876 in the March number.

